# Dietary ferrous glycinate supplementation reshapes the gut microbiota and improves intestinal barrier function in weaned piglets by reducing luminal iron accumulation

**DOI:** 10.3389/fvets.2026.1834338

**Published:** 2026-05-27

**Authors:** Yilong Zhang, Qing Gao, Conghui Yin, Yabin Wu, Dianchao Gu, Ming Huang, Dan Zhu, Daiwen Chen, Aimin Wu

**Affiliations:** 1 Key Laboratory for Animal Disease-Resistance Nutrition, Ministry of Education, Ministry of Agriculture and Rural Affairs, Key Laboratory of Sichuan Province, Animal Nutrition Institute, Sichuan Agricultural University, Chengdu, China; 2 College of Animal Science, Xichang University, Xichang, Sichuan, China; 3 Hunan Debon Biotechnology Co., Ltd., Changning, Hunan, China; 4 Tongwei Agricultural Development Co., Ltd., Chengdu, Sichuan, China

**Keywords:** ferrous glycinate, gut microbiota, intestinal barrier function, iron metabolism, piglets

## Abstract

Non-absorbed inorganic iron in the digestive tract can be directly utilized by microorganisms, particularly pathogens. This study aimed to evaluate the feasibility of substituting ferrous sulfate (FeSO₄) with lower doses of ferrous glycinate (Fe-Gly) in weaned piglets. A total of 30 weaned piglets were randomly assigned to three dietary treatments (*n* = 10): a Control group, a Gly-Fe-50 group (50 mg/kg Fe-Gly), and a Gly-Fe-75 group (75 mg/kg Fe-Gly). Compared with 100 mg/kg FeSO₄, supplementation with 50 or 75 mg/kg Fe-Gly did not significantly affect growth performance parameters, indicating that Fe-Gly maintained comparable growth performance at a 25–50% lower inclusion level. Numerically, 50 mg/kg Fe-Gly showed higher ADFI and ADG, although these differences were not statistically significant. Fe-Gly supplementation was associated with improvements in intestinal barrier function in weaned piglets. Furthermore, Fe-Gly supplementation significantly elevated serum total iron-binding capacity (TIBC) (*p* < 0.05), indicating enhanced iron transport efficiency. Gene expression and microbial sequencing analyses revealed that Fe-Gly upregulated antioxidant genes (*SLC7A11*, *P62*) in the jejunum. Additionally, it significantly augmented the proportion of beneficial microbes, such as *Lactobacillus* and *Akkermansia*, while reducing the proportion of *Proteobacteria* and *Escherichia-Shigella*. In summary, because of its high bioavailability, 50 mg/kg Fe-Gly does more than meet the growth and metabolic demands of piglets; it also reduces iron accumulation in the hindgut lumen. This mechanism restricts iron availability for intestinal pathogens, inhibiting their proliferation and thereby improving intestinal health in piglets.

## 1 Introduction

Iron is a vital trace element integral for animal development and physiological processes. Approximately 70–75% of total body iron exists in the form of heme iron; consequently, one of its primary physiological functions is to transport oxygen and carbon dioxide via hemoglobin (HGB) (1). Iron serves as a component or cofactor for numerous enzymes involved in carbohydrate metabolism, energy metabolism, and protein synthesis. Concurrently, it participates in systemic redox reactions and is closely associated with antioxidant capacity, thereby maintaining normal cellular metabolism. Furthermore, iron is widely distributed throughout the body and is essential for lymphocyte proliferation and antibody production. By regulating immune function, enhancing leukocyte activity, and inhibiting the growth of harmful bacteria, iron plays a pivotal role in the body’s immune defense system (2). In livestock production, insufficient iron intake predisposes animals to iron deficiency anemia (IDA). This condition leads to numerous adverse effects, including reduced physical activity, growth retardation, and impaired development (3). This condition is most prevalent in neonatal and weaned piglets; notably, iron deficiency during the early post-weaning period severely compromises growth performance (4, 5). Therefore, dietary supplementation with exogenous iron is a common strategy to prevent and treat iron deficiency in pigs (6).

Presently, ferrous sulfate (FeSO₄) is the most conventionally used iron source in swine production. Yet, conventional inorganic iron sources are characterized by limitations such as chemical instability and low bioavailability. Specifically, merely 5–20% of feed-derived iron consumed by piglets is localized to the intestinal tract, while the remaining 80% accumulates in the intestinal lumen, where it becomes available for utilization by microorganisms (7, 8). Consequently, when ferrous sulfate is employed as a dietary iron supplement, inclusion levels often exceed physiological requirements. This practice more than escalates feed manufacturing expenses. It also elevates the pool of iron utilizable by microbial utilization in the intestinal tract, thereby elevating the incidence of diarrhea in early-weaned piglets. Previous research has demonstrated that iron amino acid complexes offer superior absorption advantages as a supplement for piglets. Characterized by a stable chemical structure, these chelates effectively mitigate antagonistic interactions with anti-nutritional factors such as phytate, thereby significantly enhancing bioavailability and minimizing iron wastage (9, 10).

In recent years, organic amino acid-chelated iron preparations have garnered increasing attention due to their structural stability, high bioavailability, and reduced gastrointestinal irritation (11, 12). Fe-Gly, a representative of this class, effectively mitigates interference from anti-nutritional factors and exhibits superior absorption capacity compared with ferrous sulfate (13, 14). Additionally, amino acid chelated iron can regulate pH levels within the gastrointestinal tract, thereby creating favorable conditions for the efficient absorption of iron ions. Consequently, amino acid-chelated iron is widely utilized as a dietary iron supplement for weaned piglets (15). Therefore, amino acid-chelated iron, particularly Fe-Gly, is frequently utilized as an iron supplement for weaned piglets. However, to date, no studies have conclusively demonstrated that dietary iron-glycine supplements exert a positive regulatory effect on the intestinal morphology and gut microbiota of weaned piglets. Accordingly, this study aimed to investigate the effects of dietary Fe-Gly supplementation on growth performance, intestinal development, systemic iron metabolism, and gut microbiota composition in weaned piglets.

## 2 Materials and methods

All animal experiments in this study were reviewed and approved by the Animal Care and Use Committee of Sichuan Agricultural University (Chengdu, China, No.: 20210111). All mineral raw materials, including ferrous glycinate (Fe-Gly), used in this study were provided by Hunan Debon Biotechnology Co., Ltd. (Hunan, China) (Table 1).

**Table 1:** Experimental design and iron supplementation treatments.

Treatment	Diets	Number of piglets
CON	FeSO_4_ (100 mg Fe/kg)	10
Gly-Fe-50	Fe-Gly (50 mg Fe/kg)	10
Gly-Fe-75	Fe-Gly (75 mg Fe/kg)	10

### 2.1 Animals and experimental design

A total of 30 weaned piglets (21 days old; average initial body weight of 6.82 kg) were enrolled in this study. Following a 3-day acclimatization period, the piglets were weighed and allotted to three dietary treatments based on a randomized complete block design. Each treatment consisted of 10 replicates, with one pig per replicate. Based on elemental iron calculations, the experimental diets were supplemented with 100 mg /kg FeSO_4_, 50 mg/kg Fe-Gly, or 75 mg/kg Fe-Gly, respectively. During the 21-day trial, all piglets had free access to feed and water.

The experimental basal diet was formulated based on the 2012 recommendations of the NRC and meets the nutritional requirements of piglets weighing 5–11 kg (Table 2).

**Table 2:** Base diet formulation and nutritional levels (%).

Ingredient	Proportion (%)	Calculated nutrient levels	
Corn (CP7.8%)	66.100	CP (%)	17.00
Bran (CP13.6%)	4.100	EE (%)	5.85
Fishmeal (CP68%)	4.500	CF (%)	2.65
Pulped soybean meal (CP46%)	8.200	ASH (%)	5.10
Puffed soya bean (CP36.5%)	9.000	Ca (%)	0.67
Soybean oil	1.500	P (%)	0.56
Fructose	2.000	N-Phy-P (%)	0.36
Glucose	1.000	Lys (%)	1.37
Talcum powder (38%)	0.600	Met (%)	0.50
Calcium hydrogen phosphate	0.800	Cys (%)	0.25
NaCl	0.450	Thr (%)	1.00
L-Lysine hydrochloride	0.670	Trp (%)	0.28
DL-Methionine (99%)	0.210	Arg (%)	1.19
L-Threonine (98.5%)	0.405	Ile (%)	0.60
Tryptophan (98.5%)	0.105	Val (%)	0.75
Choline chloride (50%)	0.150	DE (Mcal/kg)	3.48
Phytase 5000	0.020	ME (Mcal/kg)	3.20
Antioxidants (30%)	0.020	NE (Mcal/kg)	2.45
Mildew inhibitor	0.070		
Multivitamin^a^	0.050		
Complex polymetallic^b^	0.050		
Total	100.00		

a The concentration of added vitamins per kg of diet was as follows: niacinamide, 50 mg; D-calcium pantothenate, 30 mg; VB2, 8 mg; VB12, 6 mg; VB1 and VK3, 4 mg each; folic acid, 2 mg; and biotin, 0.3 mg. The mixture also supplied 3,000 IU of VD3 and 60 IU of VE.

b Provided per kg of diet: 100 mg Fe (as FeSO₄·H₂O) in the control group and 50 mg Fe (as Fe[C₂H₄O₂N]₂) in the Fe-Gly group; plus 5.5 mg Cu ((C₂H₄NO₂)₂Cu); 3.5 mg Mn; 90 mg Zn (C₄H₈N₂O₄Zn); 0.14 mg I (Ca(IO₃)₂); and 0.275 mg Se (Na₂SeO₃).

### 2.2 Animal experiments

The animal experiment was carried out at the Teaching and Research Base of the Institute of Animal Nutrition, Sichuan Agricultural University. Prior to the experiment, comprehensive and thorough disinfection procedures were performed on the pig barns. Throughout the experiment, the pig barn maintained excellent ventilation with ambient temperatures consistently between 26 and 28 °C. Pig pens were cleaned and disinfected daily according to standard protocols. Feed was supplied three times daily at 08:00, 14:00, and 20:00. Piglet health status and feeding behavior were monitored every 2 h. To ensure ad libitum feeding, feeders were maintained with a minimum stock level of at least 25% at all times.

### 2.3 Sample harvesting and processing

#### 2.3.1 Blood sampling and processing

Upon completion of the 21-day experiment, blood was harvested from the anterior vena cava of each piglet. To facilitate whole blood assays, 1 mL was transferred into heparinized tubes (sodium heparin), while the remaining volume was placed in non-anticoagulant tubes to isolate serum via centrifugation (3,000 rpm, 15 min). The resulting serum samples were aliquoted and preserved at −20 °C pending further analysis.

#### 2.3.2 Tissue harvesting

Subsequent to blood collection, all piglets received a subcutaneous anesthetic injection prior to sacrifice via exsanguination. The abdominal cavity was rapidly opened to isolate the visceral organs and intestinal tract, and the weight of each organ was accurately measured for the calculation of organ index. Tissue specimens were either preserved in 4% PFA for histological examination (HE staining) or flash-frozen in liquid nitrogen and stored at −80 °C for genomic and proteomic assays. Simultaneously, digesta from the cecum and colon was frozen in *LN_2_* and maintained at −80 °C.

$$\text{Organ Index}\;(\%)=\frac{\text{Organ Weight}}{\text{Final Body Weight}}\times 100\%$$

### 2.4 Growth metrics

Body weight (BW) was measured at 08:00 on d 0, d 22, and the sampling day after fasting. Feed consumption was monitored by recording the amount of feed provided daily and deducting the residuals and wastage on a weekly basis. Based on these records, the Average daily gain (ADG), Average daily feed intake (ADFI) and Feed-to-gain ratio (F/G) were calculated for each stage and the overall trial period.

$$\mathrm{ADG}\;(g/d)=\frac{\text{Final}\;\mathrm{BW}\;(g)-\text{Initial}\;\mathrm{BW}\;(g)}{\text{Days}}$$

$$\text{ADFI}(g/d)=\frac{\text{Total Feed Intake}\;(g)}{\text{Days}}$$

$$F/G=\frac{\text{Total Feed Intake}\;(g)}{\text{Total Weight Gain}\;(g)}$$

### 2.5 Diarrhea score and incidence

Fecal consistency was visually assessed three times daily (at 08:00, 12:00, and 16:00) throughout the experimental period. Scoring was performed in accordance with the criteria outlined in Table 3.

$$\begin{array}{l} \text{Diarrhea rate}\;(\%)=\\ \frac{\Sigma \;(\text{Number of piglets with diarrhea}\times \text{Number of days of diarrhea})}{\text{Total number of piglets}\times \text{Total number of trial days}}\times 100\% \end{array}$$

$$\text{Diarrhea index}=\frac{\text{Total cumulative fecal score}}{\mathrm{No}.\text{of piglets}\times \text{Days of scoring}\times \text{Daily scoring frequency}}$$

**Table 3:** Criteria for fecal consistency scoring in piglets.

Fecal characteristics	Description	Score
Hard, pellet-like feces	Normal	0
Soft but formed feces	Mild	1
Pasty, unformed feces	Moderate	2
Watery, liquid feces	Severe	3

### 2.6 Intestinal histomorphology

Following fixation in 4% paraformaldehyde, jejunal specimens underwent dehydration via ascending ethanol concentrations and clearing with xylene prior to paraffin embedding. Paraffin-embedded sections were Subjected to HE staining for morphological assessment. Using a Nikon Eclipse Ci-L microscope, ten fields displaying complete and regular villus structures were captured. ImageJ analysis software was subsequently employed to quantify villus height and crypt depth.

### 2.7 Assay of non-heme iron in visceral organs and enteric segments

Approximately 30 mg of lyophilized tissue was weighed and treated with 1 mL of digestion solution (comprising 3 mol/L HCl and 0.61 mol/L trichloroacetic acid). Strict precautions were taken throughout the process to prevent exogenous iron contamination. The mixture was incubated at 65 °C for at least 50 h to ensure complete hydrolysis. During incubation, samples were vortexed three times for ≥10 min each to facilitate tissue dissociation. Post-digestion, the sample volume was adjusted to 1.5 mL and spun at 10,000 rcf for 10 min. Subsequently, 10 μL of the supernatant was transferred to a 96-well microplate and mixed with 200 μL of chromogenic working solution. Following a 10-min incubation period at ambient temperature, absorbance was measured at a wavelength of 535 nm using an automated microplate reader (16).

### 2.8 Hematological parameters

Red blood cell count (RBC), hemoglobin (HGB), hematocrit (HCT), mean corpuscular volume (MCV), and mean corpuscular hemoglobin concentration (MCHC) were determined using a fully automated hematology analyzer (Hitachi, Japan)

### 2.9 Determination of serum iron concentration

Serum iron (SI) and total iron-binding capacity (TIBC) were determined using the Pointe Scientific™ Iron/TIBC Reagent Set (Pointe Scientific, Canton, MI, United States). Unsaturated iron-binding capacity (UIBC) and transferrin saturation (TF) were subsequently calculated (16, 17).

### 2.10 Relative mRNA expression levels

The extraction of intestine total RNA was carried out by TRIzol reagent (Invitrogen, Waltham, MA, United States) according to the manufacturer’s specifications. The working solution was configured in accordance with the protocols shown in the Reverse Transcription Kit (Takara, San Jose, CA, United States). The ΔΔCT method was used to calculate relative gene expressions by collecting the cycle threshold (Ct) and normalizing it to the housekeeping gene *β-actin*.

### 2.11 16S rRNA sequencing

Total DNA from colonic content samples was extracted using the E.Z.N.A.^®^ Stool DNA Kit. Subsequently, the DNA samples were sent to Novogene Co., Ltd. (Beijing, China) for 16S rRNA gene amplicon sequencing to perform the subsequent gut microbiota analysis. Specifically, the V3-V4 hypervariable region of the 16S rRNA gene was amplified using universal primers, including the forward primer 341F (5′-CCTAYGGGRBGCASCAG-3′) and the reverse primer 806R (5′-GGACTACNNGGGTATCTAAT-3′). The amplified products were used to construct sequencing libraries with the TruSeq® DNA PCR-Free Sample Preparation Kit (Illumina, San Diego, CA, United States), and high-throughput sequencing was performed on an Illumina NovaSeq platform. Bioinformatics analysis of the sequencing data was conducted using QIIME 2 software (version 2022.2). Furthermore, based on the 16S rRNA amplicon sequences, microbial functional profiles were predicted using PICRUSt2 (Phylogenetic Investigation of Communities by Reconstruction of Unobserved States). The functional annotation in PICRUSt2 was based on multiple gene family databases, primarily including Kyoto Encyclopedia of Genes and Genomes (KEGG) Orthologs (KOs) and Enzyme Commission (EC) numbers (18, 19) (Table 4).

**Table 4:** Primer sequences used for real-time PCR.

Genes	Primer sequences (5′-3′)
β-actin	F: CACGCCATCCTGCGTCTGGA R: AGCACCGTGTTGGCGTAGAG
HAMP	F: CTGAGCGTGCAGATCCGG R: GGAGGTCTGTGAGCTGTCTT
DMT1	F: TCTTGGTGCCATCAACCCTG R: CCTCCTCAGGAATGGCGATC
TFRC	F: TGTCTTGATATACATGGACCGGG R: AGCTTTTCTGCACCAGCTCT
FTL	F: GCTCCCAGGTTCGTCAGAAT R: CGCGCTGGTTTTGCATTTTC
FTH	F: CGCGATGATGTGGCTTTGAA R: TGAAGGAAGATTCGGCCACC
FPN	F: CAGACGTCACTGGTCATCCA R: TGCTTCTGTCTTCTCCTGCA
GPX4	F: TACGTGTGCATCGTCACCAA R: TTGCAAGGGAAGGCCAGAAT
SLC7A11	F: ATGAGTGTCAGCTGGAGTGC R: GCCAGCATATGCATACATTCCA
HO-1	F: CTACACGCCCCTCTACTTCC R: AGGGCCTTCTGAGCAATCTT
NQO1	F: GGCCGAACAAAAGAAGGTGG R: CTCCCCTATGAGCACACGTT
CD98	F: TACGGAGACGAGATTGGCCT R: CACGCTGCTGTTTACAGGTC
P62	F: ATCTGTGATGGTTGCAACGG R: CTCAGAAAGGTGCCCAAAGG
NRF2	F: CTGAGACTAGCACGGTTCCA R: GAGAGGATGCTGCTGAAGGA

### 2.12 Statistical methods

Initial data management was conducted in Microsoft Excel, followed by statistical evaluation using IBM SPSS Statistics 27 (IBM Corp., Armonk, NY, United States). To assess differences across groups, a one-way ANOVA was applied with subsequent Tukey’s *post hoc* testing for pairwise comparisons. Results are presented as means ± standard error of the mean (SEM). Significance was accepted at *p* ≤ 0.05, while values between 0.05 and 0.10 were interpreted as trends. Figures were visualized using GraphPad Prism 10.1.2 (GraphPad Software, Boston, MA, United States).

## 3 Results

### 3.1 Growth performance and diarrhea frequency of piglets in response to varying dietary glycine iron concentrations

No significant differences were observed in growth performance among the treatment groups (*p* > 0.05; Table 5). Numerically, compared with the control group, piglets supplemented with 50 or 75 mg/kg Fe-Gly showed higher BW, ADFI, and ADG and lower F/G. Specifically, BW increased by 7.87 and 8.03%, ADFI by 10.36 and 12.62%, and ADG by 17.83 and 18.49% in the Gly-Fe-50 and Gly-Fe-75 groups. Furthermore, the diarrhea rate in the Gly-Fe-50 was numerically lower than that of the control group and the Gly-Fe-75.

**Table 5:** Effects of different dietary levels of Fe-Gly on growth performance and diarrhea incidence in weaned piglets.

Items	Treatment			*p*-values
	100 mg FeSO_4_	50 mg Fe-Gly	75 mg Fe-Gly	
IBW Kg	6.81 ± 0.16	6.83 ± 0.14	6.82 ± 0.13	0.995
FBW Kg	11.95 ± 0.45	12.89 ± 0.35	12.91 ± 0.62	0.300
ADFI (g/d)	371.0 ± 30.1	409.4 ± 23.8	417.8 ± 42.9	0.575
ADG (g/d)	244.9 ± 21.3	288.6 ± 18.7	290.2 ± 26.3	0.284
F/G	1.53 ± 0.05	1.43 ± 0.07	1.43 ± 0.04	0.324
Diarrhea rate/%	9.52 ± 3.33	9.05 ± 2.30	9.52 ± 3.09	0.994
Diarrhea index	0.31 ± 0.08	0.27 ± 0.06	0.30 ± 0.10	0.952

Data are presented as means ± SEM (*n* = 10). IBW, initial body weight; FBW, final body weight; ADG, average daily gain; ADFI, average daily feed intake; F/G, feed-to-gain ratio.

### 3.2 Effect of different concentrations of iron glycinate on the relative organ weights of piglets

Data presented in Table 6 indicate that the Gly-Fe-50 cohort exhibited a significantly elevated cardiac index relative to the control groups (*p* < 0.05). Concurrently, while numerical elevations in the hepatic, splenic, and renal indices were observed in the Gly-Fe-50 group versus the others, these variations lacked statistical significance.

**Table 6:** Effect of adding different concentrations of iron glycinate on piglets’ relative organ weights.

Items	Treatment			*p*-values
	100 mg FeSO_4_	50 mg Fe-Gly	75 mg Fe-Gly	
Heart index (%)	0.48 ± 0.01^b^	0.52 ± 0.01^a^	0.49 ± 0.01^ab^	0.031
Liver index (%)	2.77 ± 0.09	2.94 ± 0.04	2.8 ± 0.08	0.266
Spleen index (%)	0.21 ± 0.02	0.24 ± 0.02	0.2 ± 0.02	0.255
Lung index (%)	1.23 ± 0.04	1.24 ± 0.03	1.34 ± 0.07	0.233
Kidney index (%)	0.53 ± 0.02	0.58 ± 0.02	0.57 ± 0.02	0.226

Data are presented as means ± SEM (*n* = 10). ^a,b^denote statistical significance.

### 3.3 Effects of different iron sources on intestinal morphologic structure in piglets

**Figure 1:**
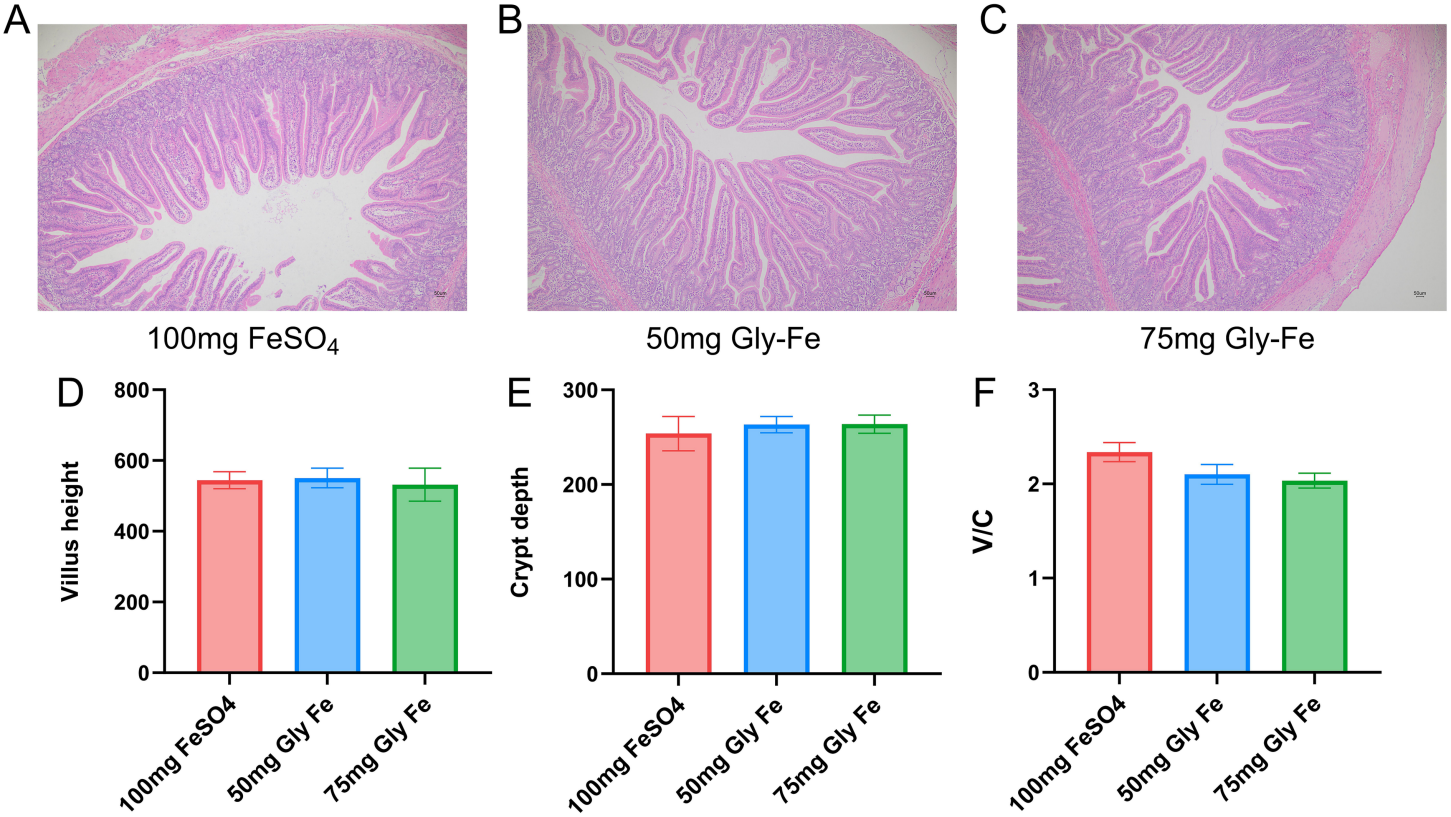
Effects of different iron sources on intestinal morphologic structure in piglets. **(A–C)** Jejunal pathological sections; **(D)** jejunal villus height, *n* = 10; **(E)** jejunal crypt depth, *n* = 10; **(F)** jejunal villus to crypt ratio, *n* = 10.

Figure 1 results indicate that comparative analysis of jejunal villus height, crypt depth, and the V/C ratio (villous height to crypt depth ratio) revealed no significant differences among the three groups (*p* > 0.05); however, the Fe-Gly groups showed a slight trend of improvement. HE staining results further confirmed that the intestinal structure in all groups was intact, with regularly arranged villi. These results indicate that supplementation with lower doses of ferrous glycinate is sufficient to achieve effects comparable to those of higher levels of FeSO_4_ and ferrous glycinate (Figures 1A–F).

### 3.4 Effects of different concentrations of iron glycinate on serum iron levels and hematological parameters in piglets

**Figure 2:**
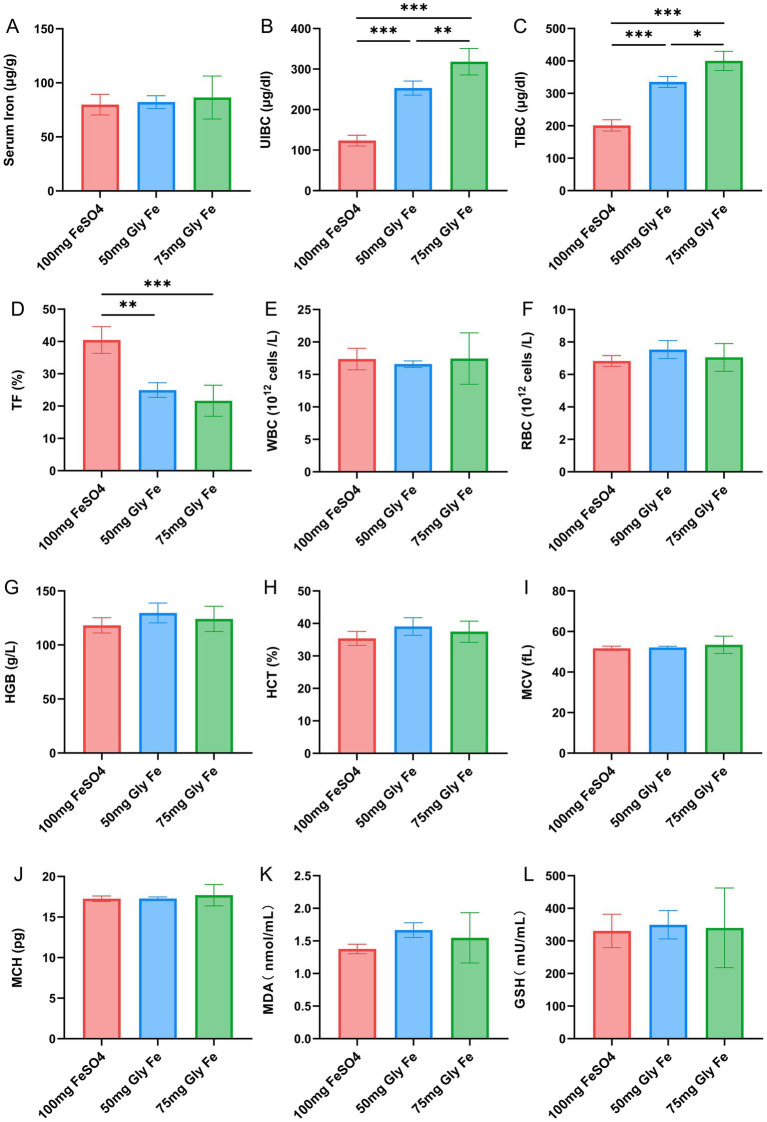
Effect of different iron sources on serum iron levels and blood routines in piglets. **(A)** Serum iron, *n* = 10; **(B)** unsaturated iron-binding capacity, *n* = 10; **(C)** total iron-binding capacity, *n* = 10; **(D)** transferrin saturation, *n* = 10; **(E)** leucocyte number, *n* = 10; **(F)** erythrocyte number, *n* = 10; **(G)** hemoglobin, *n* = 10; **(H)** erythrocyte pressure area, *n* = 10; **(I)** mean corpuscular volume, *n* = 10; **(J)** mean cell hemoglobin, *n* = 10; **(K)** MDA level in the serum, *n* = 10; **(L)** GSH level in the serum, *n* = 10.

As presented in Figure 2, serum iron levels (Figure 2A) did not differ significantly among the groups. However, compared with the control group, Fe-Gly supplementation significantly increased UIBC and TIBC (*p* < 0.05; Figures 2B,C) but significantly decreased transferrin saturation (TF) compared with the control group (*p* < 0.05; Figure 2D). Furthermore, these relevant parameters showed a positive correlation with the dosage of Fe-Gly supplementation. There were no significant intergroup differences in the remaining hematological parameters (Figures 2E–J) and serum GSH and MDA concentrations (Figures 2K,L).

### 3.5 Effects of different concentrations of iron glycinate on iron metabolism in the body

**Figure 3:**
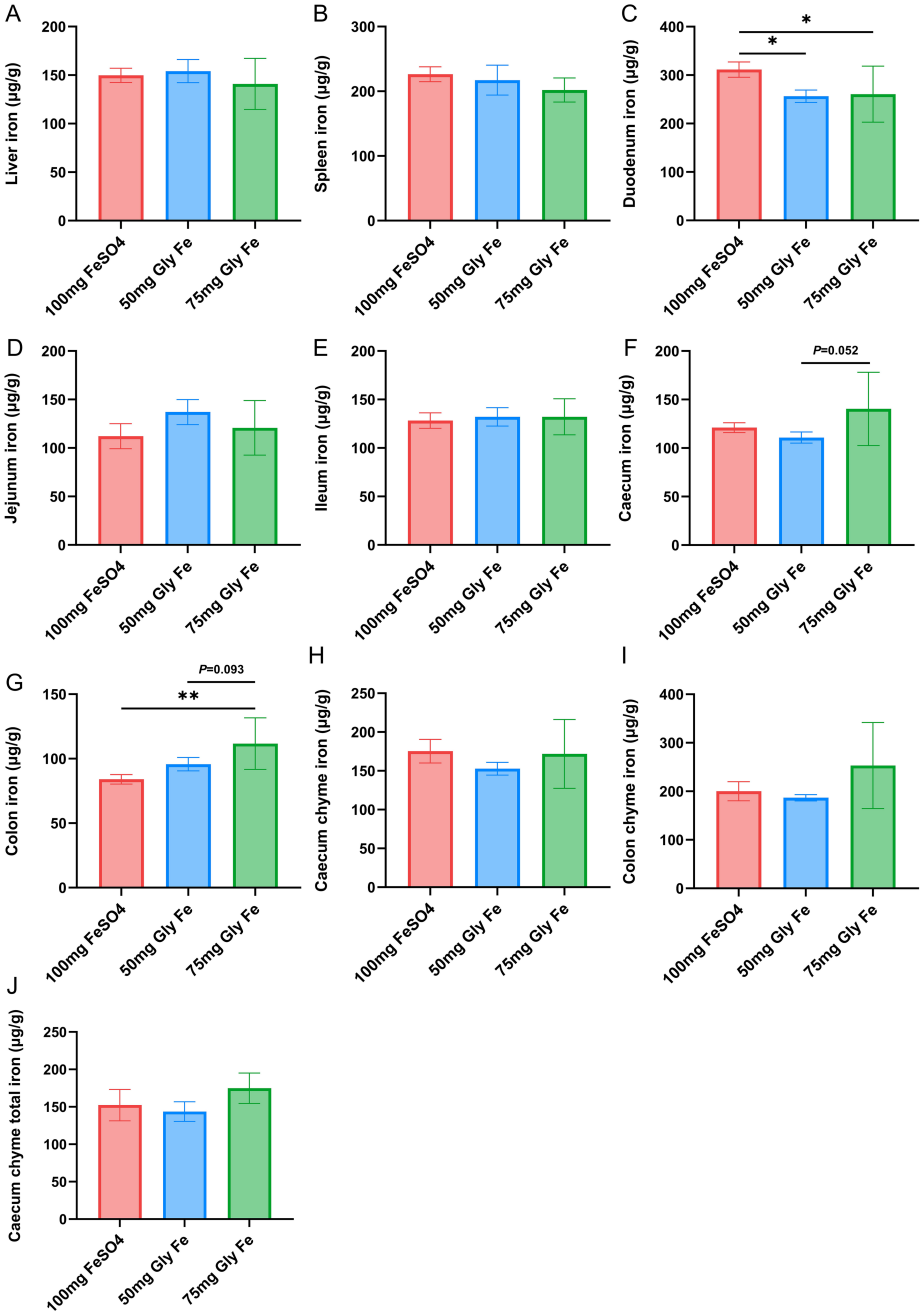
Effects of different concentrations of iron glycinate on iron metabolism in the body. **(A)** Liver iron, *n* = 10; **(B)** spleen iron, *n* = 10; **(C)** duodenum iron, *n* = 10; **(D)** jejunum iron, *n* = 10; **(E)** ileum iron, *n* = 10; **(F)** caecum iron, *n* = 10; **(G)** colon iron, *n* = 10; **(H)** caecum chyme iron, *n* = 10; **(I)** colon chyme iron, *n* = 10; **(J)** caecum chyme total iron, *n* = 10.

As shown in Figure 3, compared with the control group, the duodenal tissue iron content in the Fe-Gly groups was significantly decreased (*p* < 0.05, Figure 3C), whereas the colonic tissue iron content was significantly increased (*p* < 0.01, Figure 3G). Additionally, cecal tissue iron content exhibited an upward trend (*p* = 0.052, Figure 3F). The findings of the detection of iron residues in the cecal and colonic segments digesta showed that the iron residue levels in the Gly-Fe-50 group were lower than those in the control group and the Gly-Fe-75 group (Figures 3H–I).

### 3.6 Modulation of jejunal iron metabolism-related gene expression by graded levels of glycine iron in piglets

**Figure 4:**
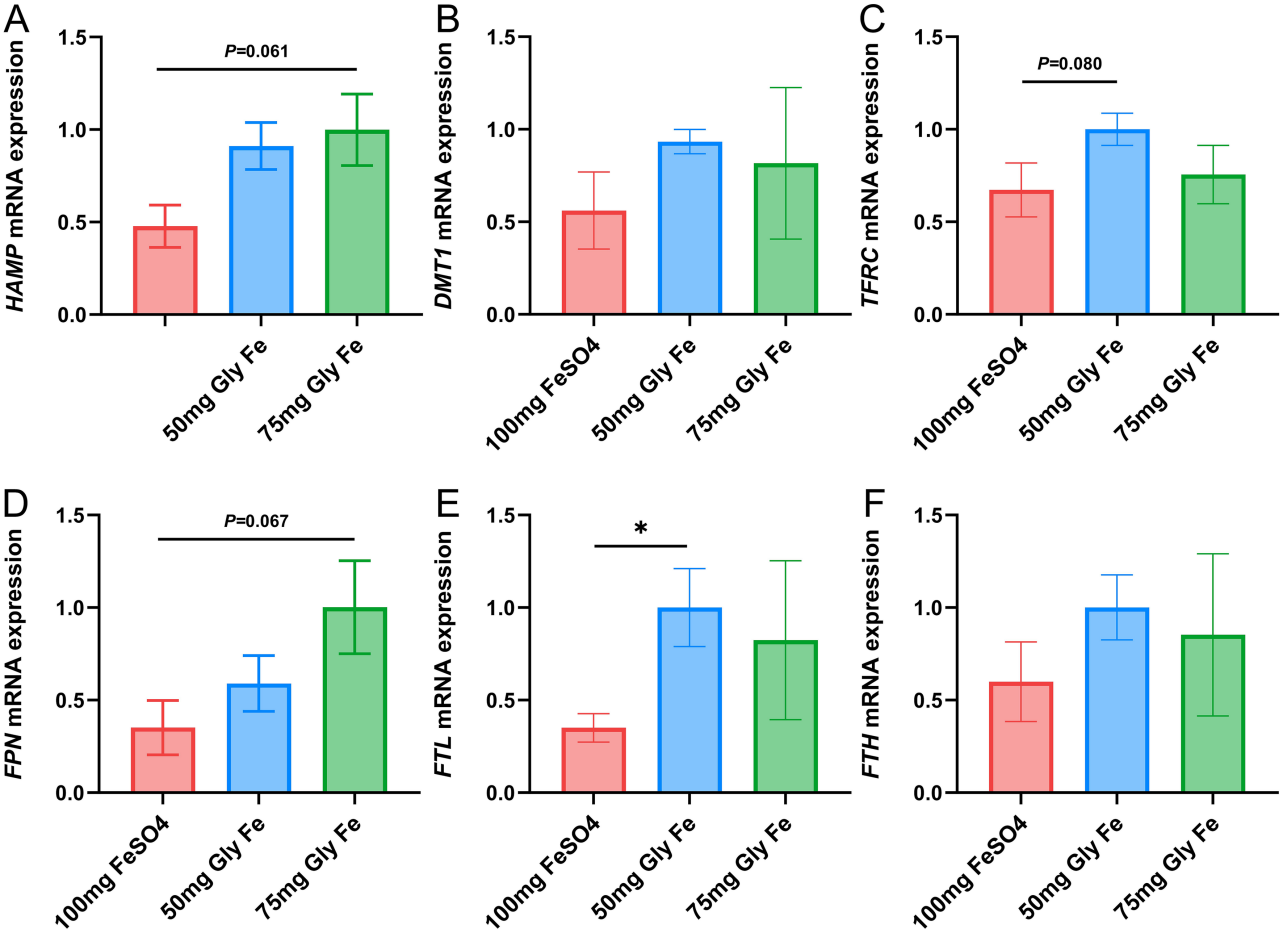
Modulation of iron metabolism-related gene expression in the small intestine of piglets by graded levels of glycine iron. **(A)** Hepcidin antimicrobial peptide (*HAMP*), *n* = 10; **(B)** divalent metal transporter 1 (*DMT1*), *n* = 10; **(C)** transferrin receptor C (*TFRC*), *n* = 10; **(D)** ferroportin (*FPN*), *n* = 10; **(E)** ferritin light chain (*FTL*), *n* = 10; **(F)** ferritin heavy chain (*FTH*), *n* = 10.

As illustrated in Figure 4, a tendency toward increased *TFRC* expression was observed in the Gly-Fe-50 group (*p* = 0.080; Figure 4C). Concurrently, the Gly-Fe-50 group significantly upregulated the transcriptional level of the *FTL* gene in jejunal tissue relative to the control (*p* < 0.05; Figure 4E). The expression of other genes was numerically elevated, but the differences were not significant (Figures 4D,F). In contrast, although the Gly-Fe-75 also increased the transcript levels of iron metabolism-related genes (*DMT1*, *TFRC*, *FTL*, and *FTH*) in the jejunum, the effect was less pronounced than that of the Gly-Fe-50 (Figures 4B,C,E,F). These findings suggest that a low dose of Fe-Gly is sufficient to enhance intestinal iron absorption and maintain systemic iron homeostasis in piglets.

### 3.7 Modulation of antioxidant-related gene expression in the jejunum of piglets by graded levels of glycine iron

**Figure 5:**
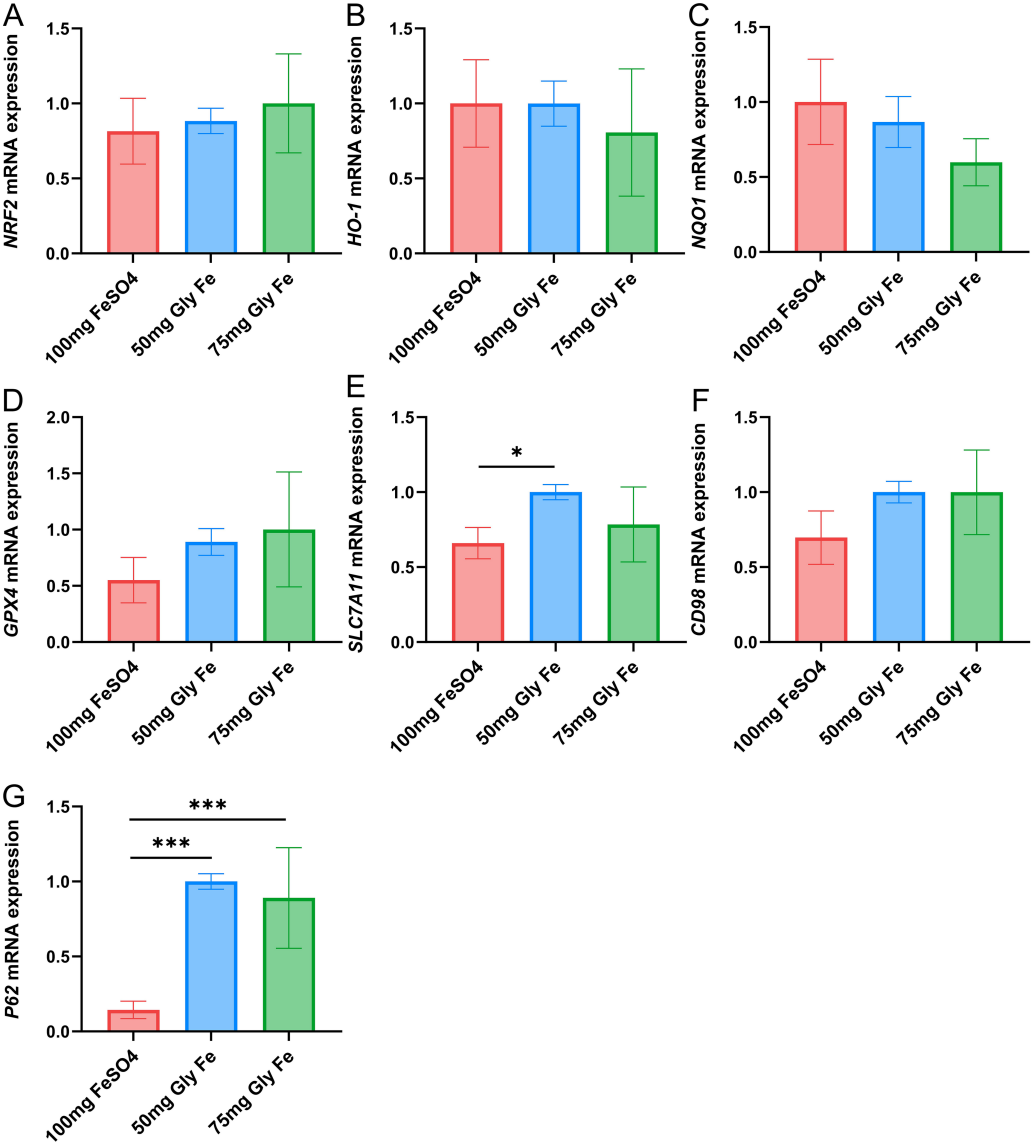
Effects of different concentrations of iron glycinate on the expression of antioxidant-related genes in the jejunum of piglets. **(A)** Nuclear factor erythroid 2-related factor 2 (*NRF2*), *n* = 10; **(B)** Heme oxygenase-1 (*HO-1*), *n* = 10; **(C)** NAD(P)H quinone oxidoreductase 1 (*NQO1*), *n* = 10; **(D)** Glutathione peroxidase 4 (*GPX4*), *n* = 10; **(E)** Solute carrier family 7 member 11 (*SLC7A11*), *n* = 10; **(F)** Cluster of differentiation 98 (*CD98*), *n* = 10; **(G)** Sequestosome 1 (*P62*), *n* = 10.

As illustrated in Figure 5, the Gly-Fe-50 group significantly upregulated the expression of *SLC7A11* (*p* < 0.05; Figure 5E), *P62* (*p* < 0.001; Figure 5G) within the jejunal segment in comparison with the control. The mRNA abundance of *GPX4*, *CD98* also exhibited an upward trend (Figures 5D,F), although these differences did not reach statistical significance. In contrast, although group Gly-Fe-75 exhibited similar gene expression trends, no significant dose-dependent enhancement was observed. Concurrently, there were no significant differences in the mRNA expression levels of *NRF2*, *HO-1*, or *NQO1* among the treatment groups (*P* > 0.05).

### 3.8 Impact of distinct iron sources on the compositional profile and functional potential of the intestinal microbiota in piglets

**Figure 6:**
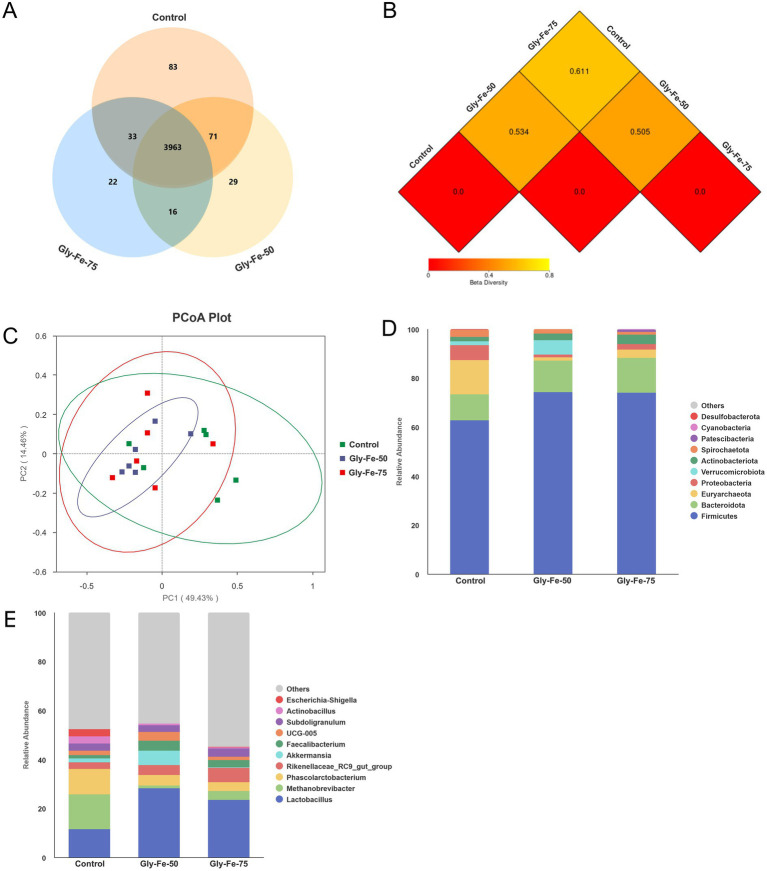
Effects of different iron sources on intestinal microbial composition and function in piglets. **(A)** Functional prediction based on PICRUSt2; **(B)** heatmap of beta diversity; **(C)** beta diversity analysis based on PCoA; **(D)** phylum level in hindgut chyme; **(E)** genus level genera in hindgut chyme.

The effects of Fe-Gly on the gut microbiota were evaluated through 16S rRNA high-throughput sequencing of hindgut digesta. PICRUSt2 functional prediction analysis (Figure 6A) revealed that while core functional gene distributions were highly consistent across treatment groups, Fe-Gly treatment was associated with the enrichment of specific functional pathways, suggesting that it may confer potential biological advantages in metabolic regulation. Further analysis at the community structure level revealed that β-diversity heatmaps (Figure 6B) showed significant alterations in microbial structure for both Fe-Gly doses compared to the control group. Notably, the distance between the control group and the Gly-Fe-50 group was greater than that between the control and Gly-Fe-75 groups, suggesting that the lower dose of Fe-Gly was associated with a more distinct shift in overall microbial community structure. However, Beta diversity analysis based on PCoA (*P* > 0.05, Figure 6C) revealed no statistically significant shifts in the overall microbial community composition among the treatment groups.

Regarding taxonomic composition, Fe-Gly supplementation significantly optimized the microbial structure. At the phylum level (Figure 6D), Fe-Gly promoted the proliferation of beneficial phyla, such as *Bacteroidetes* and *Spirochaetes*. Further genus-level analysis (Figure 6E) showed a marked increase in the abundance of probiotics, including *Lactobacillus* and *Akkermansia*, alongside a decrease in potential pathogens like *Escherichia*.

## 4 Discussion

Weaned piglets are highly susceptible to stress reactions when undergoing environmental transitions and dietary adjustments, while also commonly facing the risk of iron deficiency. On one hand, the iron content in sow’s milk is inherently low; on the other hand, newborn piglets possess extremely limited iron reserves in their livers. This results in the iron obtained through natural pathways being far insufficient to support the rapid growth and development demands of piglets. Consequently, to ensure healthy development, exogenous iron supplementation via injection is typically required within 3 days of birth. After weaning, the prevention of iron deficiency in piglets is primarily achieved by supplementing their diets with highly bioavailable sources of iron.

Ma et al. (20) found that dietary supplementation with 200 mg/kg of Fe-Gly significantly increased the average daily gain (ADG) and average daily feed intake (ADFI) of weaned piglets, and effectively alleviated diarrhea during the early post-weaning period. Sun et al. (21) demonstrated that dietary supplementation with 100 mg/kg of Fe-Gly significantly improved the feed conversion ratio, enhanced immune function, and promoted intestinal health in weaned piglets. Meng et al. (22) found that oral administration of Fe-Gly effectively increased the weaning weight of piglets at 21 days of age and elevated hemoglobin and serum iron levels, thereby improving iron absorption and utilization. In the present study, supplementation with Fe-Gly improved the ADG and ADFI, reduced the F/G, and alleviated diarrhea in piglets (Table 5). Although these indices did not reach statistical significance, the observed trends are consistent with previous research findings. Notably, compared with the Gly-Fe-50, the Gly-Fe-75 did not further improve growth performance and anti-diarrheal efficacy. The findings of the present study indicate that supplementing the diet with 50 mg/kg Fe-Gly is sufficient to meet the iron requirements of weaned piglets and exerts a positive effect on their growth performance and developmental progress. Consistent with the observed growth trends, the analysis of relative organ weights (Table 6) demonstrated that the heart index was significantly higher in piglets supplemented with 50 mg/kg Fe-Gly than in those receiving 100 mg/kg FeSO_4_. As the central organ of the circulatory system, an elevated heart index indicates enhanced myocardial development and robust cardiovascular function, which are essential for sustaining the high metabolic rates required during the rapid growth phase of weaned piglets (23). We postulate that the unique molecular structure and distinctive absorption pathways of Fe-Gly facilitate more efficient intestinal uptake and subsequent transport to cardiac tissue. This likely promotes intramyocardial iron utilization and myoglobin synthesis, ultimately manifesting as a significant increase in the heart index and superior physiological efficacy compared to high-dose inorganic iron. Fe-Gly is formed by the coordination of one ferrous ion with two glycine molecules via coordinate covalent bonds, resulting in a stable chelated ring structure. This configuration may reduce its interaction with antinutritional factors such as phytate and phosphate, thereby improving iron bioavailability. In addition, the stable chelated form may limit the availability of free iron to participate in the Fenton reaction, potentially lowering the risk of oxidative damage in the intestinal environment (24). Iron in FeSO₄ is primarily absorbed via the divalent metal ion transporter pathway, whereas Fe-Gly may participate in absorption through amino acid or small peptide transport systems, a process relatively less influenced by intestinal environmental factors (25).

Iron within the body is primarily transported in the blood in a form bound to transferrin. Total iron-binding capacity serves as a crucial indicator reflecting plasma transferrin levels and its iron-binding capacity. Elevated TIBC typically suggests enhanced iron transport potential within the body (26). Additionally, transferrin saturation (Tf) reflects the proportion of serum iron bound to transferrin, making it a key parameter for assessing the balance between iron supply and demand within the body. In this study, both UIBC and TIBC were significantly elevated in the Fe-Gly treatment group, suggesting improved iron transport and distribution capacity (27). Concurrently, Tf in the Fe-Gly group decreased significantly, indicating that iron could be transported and utilized more efficiently rather than remaining in the circulatory system. This facilitates the maintenance of relatively stable iron homeostasis in the body. In summary, under conditions of 25–50% reduced iron supplementation, Fe-Gly maintains stable blood iron homeostasis and normal hematopoietic function while moderately enhancing iron transport efficiency. This provides important physiological evidence supporting the replacement of high-dose FeSO₄ with Fe-Gly.

An intriguing finding of the present study was the divergent distribution of iron content along the intestinal tract: Compared with the control group, Fe-Gly supplementation was associated with lower iron concentrations in the duodenum but higher levels in the colon. Although this pattern appears inconsistent with the conventional view that organic trace minerals are primarily absorbed in the proximal small intestine (28), it may instead reflect more dynamic absorption and metabolic processes. The reduced duodenal iron content may indicate enhanced absorption efficiency, as the relatively high bioavailability of Fe-Gly could facilitate rapid uptake by enterocytes via transporters such as DMT1 and subsequent export into circulation through ferroportin (29), leaving less residual iron in the mucosa at sampling. Conversely, the increased colonic iron content may be linked to interactions with the hindgut microbiota. Iron escaping complete absorption in the small intestine may reach the distal gut, where it becomes available to resident microbes that require iron for growth and metabolic activity (30). In addition, the physicochemical properties of the chelated form may influence its regional behavior, as Fe-Gly could interact differently with mucins or luminal components in the colon compared with inorganic iron sources (31). Together, these findings suggest that Fe-Gly supplementation may involve coordinated host–microbiota interactions that shape regional iron distribution along the intestinal tract.

Dietary iron is primarily transported into intestinal epithelial cells (enterocytes) via divalent metal transporter 1 (*DMT1*) in the duodenum and proximal jejunum. While the duodenum contains high levels of DMT1, its capacity is constrained by a short transit time. Conversely, the jejunum, with its extensive absorptive surface area and longer retention time, is physiologically responsible for the bulk of nutrient absorption. Subsequently, it is released into the bloodstream via the basolateral transporter ferroportin (*FPN*) and transported to the liver for storage (32). Further iron content analysis revealed that duodenal iron levels in both Fe-Gly treatment groups were significantly lower than those in the control group. In contrast, the iron content in the jejunum of the Gly-Fe-50 treatment group was higher than that in the other two groups. Given that pathogenic bacteria, such as *Escherichia coli*, primarily colonize the jejunum and ileum, we further evaluated the mRNA abundance of iron metabolism-related genes iron metabolism within the jejunal tissue to investigate the impact of Fe-Gly on this intestinal segment. The outcomes from this trial indicate that supplementing the diet with 50 mg/kg Fe-Gly tended to upregulate DMT1 and TFR1 gene expression in the jejunum. While these numerical increases require further validation, they align with the premise of Fe-Gly’s efficient iron absorption. Furthermore, the upregulation of FPN facilitated the transport of intracellular iron into the circulation, thereby improving systemic iron bioavailability (33); The increased expression of FTL and FTH (Figure 4) facilitates the safe storage of excess intracellular iron, thereby preventing oxidative damage induced by the excessive accumulation of free iron (34, 35).

Ferroptosis is a regulated form of cell death characterized by the accumulation of iron-dependent lipid peroxides. Systemic iron overload may trigger ferroptosis, thereby causing damage to cells such as intestinal epithelial cells (36). *GPX4* is a potent intracellular antioxidant enzyme responsible for catalyzing the reduction of lipid hydroperoxides to lipid alcohols, thereby protecting cells from oxidative damage (37). *SLC7A11* serves as the catalytic subunit of the cystine/glutamate antiporter. By facilitating the uptake of extracellular cystine, it provides essential precursors for the synthesis of the antioxidant glutathione (*GSH*). *CD98* forms a heterodimer with *SLC7* family transporters, and together, they function synergistically to maintain cellular antioxidant capacity and defend against oxidative damage (38, 39). The outcomes obtained from this trial indicate that supplementing piglet diets with 50 mg/kg Fe-Gly effectively enhances the expression levels of the *GPX4* and *CD98* genes in jejunal tissue and significantly upregulates the *SLC7A11* and *P62* genes. Interestingly, NQO1 expression trended downward in the Fe-Gly groups. We hypothesize this is attributed to reduced baseline oxidative stress in the intestine. Unlike ferrous sulfate, which readily releases free iron to catalyze ROS-generating Fenton reactions, the stable chelated structure of Fe-Gly restricts free iron release and mitigates ROS overproduction. Consequently, this diminished oxidative stress in the jejunum likely reduced the compensatory activation of NQO1, a typical stress-inducible detoxification enzyme, leading to its relative downregulation.

Firmicutes and Bacteroidetes are the dominant phyla in the gut microbiota (40). Both play a pivotal role in carbohydrate metabolism and the synthesis of short-chain fatty acids (SCFAs), making them crucial for maintaining intestinal barrier function and host energy metabolism (41). In contrast, Proteobacteria encompasses a variety of pathogenic bacteria. An increase in its relative abundance often exerts adverse effects on livestock and poultry, such as compromising the structural integrity of the intestinal barrier and synergizing with other pathogens to inhibit the proliferation of beneficial bacteria, thereby further disrupting the homeostasis of the gut microbiota. Dong et al. (42) reported that Fe-Gly could suppress the expansion of Proteobacteria while increasing the proportions of Firmicutes and Bacteroidetes, thereby contributing to improved intestinal health and enhanced nutrient digestion and absorption efficiency in piglets. Consistent with previous findings, the present study, based on phylum-level analysis of 16S rRNA sequencing, further confirmed the regulatory role of Fe-Gly: supplementation significantly promoted the proliferation of beneficial bacteria, such as Firmicutes, Bacteroidetes, and Verrucomicrobia, while effectively inhibiting the abundance of potential pathogens, including *Proteobacteria* and *Euryarchaeota*. However, it should be noted that the overall microbial community structure was not fundamentally altered, and the observed differences were primarily reflected in shifts in the relative abundance of specific taxa rather than in a complete restructuring of the gut microbiota.

Further analysis at the genus level revealed that Fe-Gly effectively increased the relative abundance of beneficial bacteria, such as *Lactobacillus* and *Akkermansia*, while concurrently reducing the relative abundance of *Escherichia*. As a representative probiotic genus within the phylum Firmicutes, *Lactobacillus* possesses diverse physiological functions. First, it can inhibit the colonization of pathogens by secreting bacteriocins and lowering intestinal pH, thereby assisting the host in establishing a microbial barrier (43); Secondly, it promotes the secretion of intestinal immunoglobulins and activates immune cells, such as macrophages and dendritic cells, thereby enhancing the immune capacity of the animal (44). Finally, through fermentation, it decomposes complex carbohydrates into short-chain fatty acids (SCFAs). Concurrently, it stimulates intestinal epithelial cells to secrete increased levels of digestive enzymes, thereby improving the digestion and absorption efficiency of the diet (45). *Akkermansia* is considered a hallmark bacterium of intestinal health, capable of enhancing intestinal barrier function through competitive exclusion and the promotion of mucosal repair. These results indicate that dietary supplementation with Fe-Gly modulates the structure of the intestinal microbial community, effectively promoting the proliferation of beneficial bacteria while suppressing the population density of pathogens. Based on this, we believe that the high bioavailability of Fe-Gly is the core factor enabling its diverse beneficial effects. As an organic iron source, it is efficiently absorbed and utilized by the intestine, thereby reducing luminal iron accumulation and restricting the availability of iron for pathogenic bacteria.

Previous research indicates that beneficial bacteria, such as *Lactobacillus*, have substantially lower iron requirements compared to pathogens like *Escherichia coli* (*E. coli*) and *Salmonella* (46). These beneficial bacteria are capable of effectively acquiring essential nutrients under iron-restricted conditions through their specific metabolic activities. Consequently, they can sustain their growth and reproduction rates even when intestinal iron availability is limited (47). Conversely, iron is an indispensable element for pathogens, such as *Escherichia coli*, as it is required for cellular respiration and key enzymatic reactions. Consequently, in an iron-restricted intestinal environment, their growth and proliferation are severely inhibited (46). The differential adaptability of bacteria regarding iron requirements provides a compelling explanation for the findings of this study: specifically, in the Fe-Gly -supplemented groups, the relative abundance of beneficial bacteria, such as *Lactobacillus* and *Akkermansia*, increased, whereas the relative abundance of harmful bacteria, such as *Escherichia coli*, decreased (Figure 6). Furthermore, analysis of the hindgut digesta revealed that the residual iron content in the Gly-Fe-50 was lower than that in the other two groups (Figure 3). This phenomenon aligns with the concept of “nutritional immunity,” wherein the host sequesters iron to restrict the growth of pathogenic bacteria. These alterations in the microbial community structure provide indirect evidence of the superior absorption efficiency of Fe-Gly, thereby underscoring the advantage of dietary supplementation with of Fe-Gly.

## 5 Conclusion

In the present study, the growth performance of piglets supplemented with 50 mg/kg Fe-Gly did not differ significantly from that of the control group. This demonstrates that Fe-Gly can effectively replace ferrous sulfate at a reduced iron inclusion level without compromising the growth performance of weaned piglets. Furthermore, by modulating the gut microbiota, Fe-Gly promoted the proliferation of beneficial microbes (such as *Lactobacillus* and *Akkermansia*) while suppressing pathogens (such as *Escherichia coli*). Additionally, Fe-Gly enhanced the expression of antioxidant-related genes in the jejunum, suggesting potential improvements in oxidative status. The high bioavailability of Fe-Gly played a central role in these benefits, as its efficient intestinal absorption minimized luminal iron residues, thereby helping to maintain intestinal health.

## Data Availability

The data presented in the study are deposited in the NCBI Sequence Read Archive (SRA) repository, accession number PRJNA1467219.
